# Case Report: A rare case of early esophageal basaloid squamous cell carcinoma with ductal differentiation

**DOI:** 10.3389/fonc.2025.1508285

**Published:** 2025-02-28

**Authors:** Xing Qi, Zhenxiang Zuo, Guangchun Li, Xiujie Cui, Honglei Wu

**Affiliations:** ^1^ Department of Gastroenterology, The Second Hospital, Cheeloo College of Medicine, Shandong University, Jinan, Shandong, China; ^2^ Department of Hematology, The Second Hospital, Cheeloo College of Medicine, Shandong University, Jinan, Shandong, China; ^3^ Department of Pathology, The Second Hospital, Cheeloo College of Medicine, Shandong University, Jinan, Shandong, China

**Keywords:** early esophageal carcinoma, EBSCC, ductal differentiation, IPCL, submucosal infiltration

## Abstract

**Background:**

Early esophageal basaloid squamous cell carcinoma (EBSCC) with ductal differentiation is a rare variant of esophageal cancer (EC), for which the clinical behavior, endoscopic features and diagnostic criteria are not fully elucidated.

**Case presentation:**

An 81-year-old female presented to our hospital with a year of eating obstruction sensation. A rough, superficial-elevated and circumferential 1/2 esophageal lesion on the upper esophagus was found by the white light endoscopy (WLE). The uplift and swelling caused by subepithelial lesions suggest that this lesion may infiltrate into the submucosa. Under the narrow band imaging magnifying endoscopy (NBI-ME), papillary-like structures were visible in the lesion, and the loop capillaries classified as intraepithelial papillary capillary loop (IPCL) of type B1 were observed. Furthermore, a fine network pattern of microvascular similar to the surface glandular structure in early differentiated gastric cancer can be seen in the middle of type B1, indicating that the presence of special glandular tubular-like components. Microscopically, the tumor was a poorly differentiated EBSCC with a glandular structure. In addition, the tubular structures transitional with the surface squamous cell carcinoma. Immunohistochemical results showed that the bilayer-tubular structures exhibited bidirectional differentiation towards the outer basal epithelium and the inner columnar epithelium. The double-layer epithelium cells of the tumorous tubules expressed CK7, and the outer cells expressed p40, CK5/6.

**Conclusion:**

Early EBSCC with ductal differentiation has unique endoscopic, histological and immunophenotypic characteristics. Understanding of the endoscopic and pathological features of the early EBSCC with ductal differentiation is crucial for early detection, accurate diagnosis, timely treatment, and prevention of misdiagnosis.

## Introduction

Esophageal cancer (EC) is a prevalent form of cancer and is also the most common cause of cancer-related deaths globally (1, 2). It primarily consists of two major subtypes: esophageal squamous cell cancer (ESCC) and esophageal adenocarcinoma (EAC). ESCC accounts for more than 90% of all cases worldwide, and has a higher incidence in East Asia (3).

Early esophageal cancer (EEC) is defined that the neoplastic lesions limited to the mucosa or submucosa regardless of the nodal status (4). Endoscopic resection (ER) is preferred treatment for EEC medically suitable patients. Compared to esophagectomy, ER has the characteristics of less trauma and lower complications. Early detection and treatment are key to improving the survival rate and the quality of life of patients with EC.

Esophageal basaloid squamous cell carcinoma (EBSCC) is a rare and poorly differentiated variant of ESCC. In the fifth edition of the WHO Classification of EC, EBSCC is classified as a subtype of ESCC (5). The cytological and histological characteristics of EBSCC are similar to those of normal squamous epithelial basal cells, but with a lower differential degree and worse prognosis. In addition, EBSCC has various histological types, such as solid nests with or without comedonecrosis, ductal differentiation, microcystic formation and hyaline-like material deposition. However, the mutational profiles of EBSCC and possible therapeutic targets have not yet been adequately examined.

EC with ductal differentiation is extremely rare. Endoscopic manifestations of ductal differentiation are most superficial mucosal erosion, ulcers, and luminal stenosis. Pathologically, this type of tumor presents a glandular-like morphology and a bilayer epithelial structure, which includes the inner luminal epithelium and the outer basal epithelium. Moreover, the tumor may accompany with adenocarcinoma or squamous cell carcinoma components (6, 7). At present, the origin and specific regulatory mechanisms of EC with ductal differentiation have not been comprehensively evaluated. Histologically, the glandular structure also grows beneath the overlying surface epithelium and prone to infiltrate into the deep layer of esophageal. It is often difficult to predict the infiltration depth under endoscopy. As a result, these tumors are often detected at advanced stages.

Here, we report a case of early EBSCC with ductal differentiation, it has unique endoscopic and pathological features. Under white light endoscopy (WLE), an uplift and swelling focus can be seen in the center of the submucosal lesion. In addition, a fine network pattern of microvascular, resembling gastric papillary adenocarcinoma can be observed under the narrow band imaging magnifying endoscopy (NBI-ME). Postoperative pathology showed that the tumor center was poorly differentiated EBSCC with glandular tubular-like structures. Although this case is an early EC, it is accompanied with poorly differentiated EBSCC components and infiltrated into the deep layer of submucosa, indicating a poor prognosis. In this study, we described the endoscopic, morphological and the immunophenotypic features of an early EBSCC with ductal differentiation, and in order to help endoscopists and pathologists improve the understanding of such lesions.

## Case report

An 81-year-old female presented to our hospital for treatment with a year of eating obstruction sensation and occasionally with acid reflux. The patient was healthy before, have no history of smoking, drinking or other unhealthy habits and no remarkable past or familial history. Both the physical and the laboratory examinations revealed no abnormality. However, the chest enhanced CT showed a thickening of the esophageal wall.

**Figure 1:**
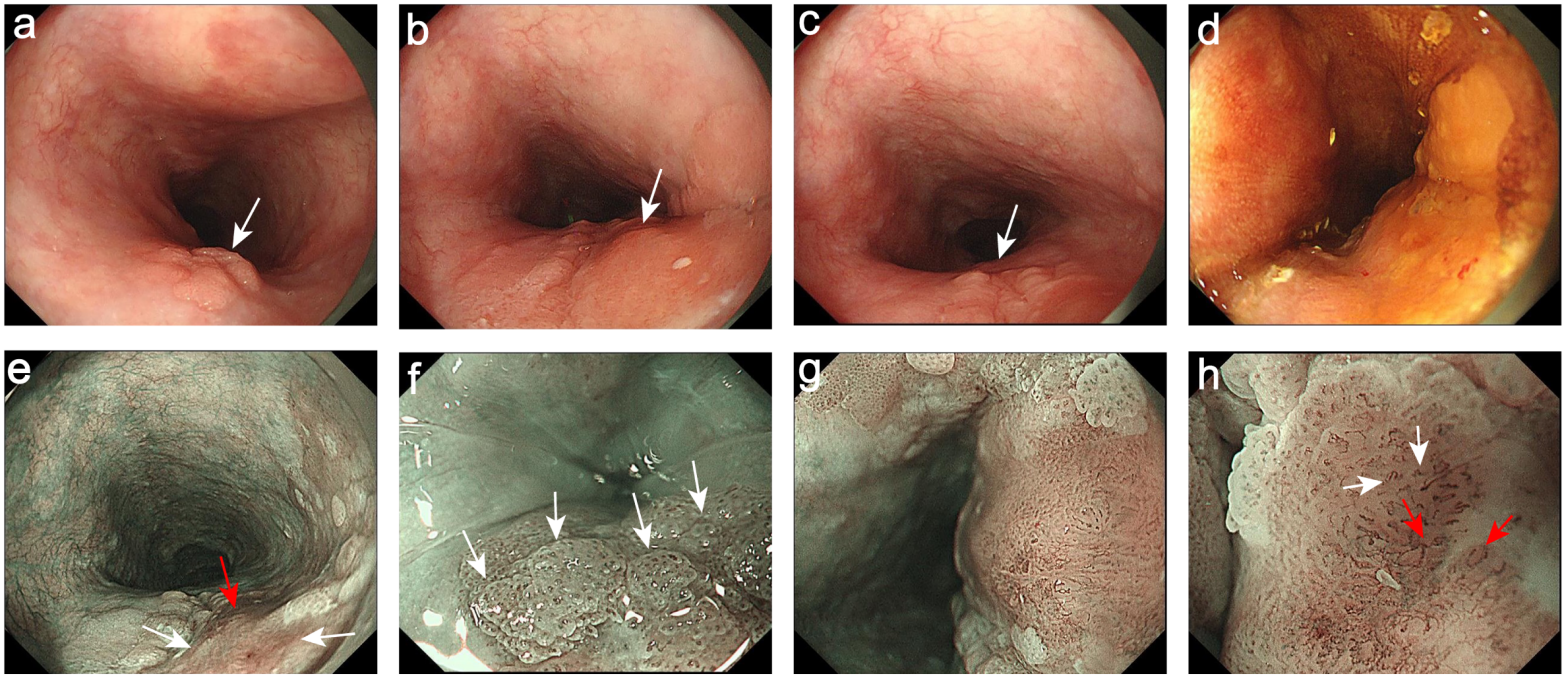
Endoscopic images. **(a)** The oral side of the lesion was light-red and presented a fine-grain sensation (White arrow). **(b)** The central dark red colored area was the uplift and swelling focus (White arrow). **(c)** The anal side of the lesion was light-red and presented a fine-grain sensation (White arrow). **(d)** The lesion was unstained with Lugol’s iodine. **(e)** The NBI showed the margin areas were light-brown (White arrows) and the central areas were dark brown (Red arrow). **(f)** The NBI-ME showed the papillary-like structures (White arrows). **(g)** The NBI-ME showed that the fine network pattern of microvascular can be observed in the middle of the IPCL of type B1. **(h)** The magnifying of **(g)**: The fine network pattern of microvascular (Red arrows) and the IPCL of type B1 (White arrows).

Then the patient underwent the upper gastrointestinal endoscopy. The white light endoscopy (WLE) showed a superficial-elevated (Type 0-IIa) lesion in the upper esophagus 19-22 cm from the incisor teeth. The lesion was patchy-reddish, rough-surface, irregular-shape and involving in circumferential 1/2 esophageal within a clear boundary. Further observation revealed that the color of the lesion mucosa was heterogeneity, with a light red margin and a deep red central. In addition, the surface of the light-red colored areas on the oral and anal sides of the lesion presented a fine-grain sensation (**Figures 1a, c**). The surface of the central deep red colored area is slightly-rough, and there is an uplift and swelling caused by the subepithelial lesion (**Figure 1b**), suggesting this lesion may infiltrate into the submucosa. Under the narrow-band imaging (NBI), the lesion was brown in the whole and light-brown in the margin area, as well as dark brown in the central area (**Figure 1e**). Under the magnifying endoscopy with NBI (NBI-ME), papillary-like structures were visible in the light-brown area (**Figure 1f**) and the loop capillaries classified as intraepithelial papillary capillary loop (IPCL) of type B1 were observed. Different from the common vascular pattern of superficial esophageal squamous cell cancer (ESCC), IPCL of type B1 were also seen at the edges of the central dark-brown region. Also, a fine network pattern of microvascular similar to the surface glandular structure in early differentiated gastric cancer can be seen in the middle of type B1 (**Figures 1g, h**), indicating the presence of special glandular tubular-like components. At the same time, the lesion was unstained with Lugol’s iodine (**Figure 1d**).

Then, the pathological diagnosis of the biopsy specimen was esophagus squamous high-grade intraepithelial neoplasia, suspected of regional infiltration. We had fully communicated with the patient’s family and informed them of the specific situation. Considering the older age of the patient, large trauma and the high risk of radical surgery, they chose to perform the palliative endoscopic submucosal dissection (ESD). The tissue was removed and sent to pathology, and the size of the lesion was about 3.5×3.0 cm.

**Figure 2:**
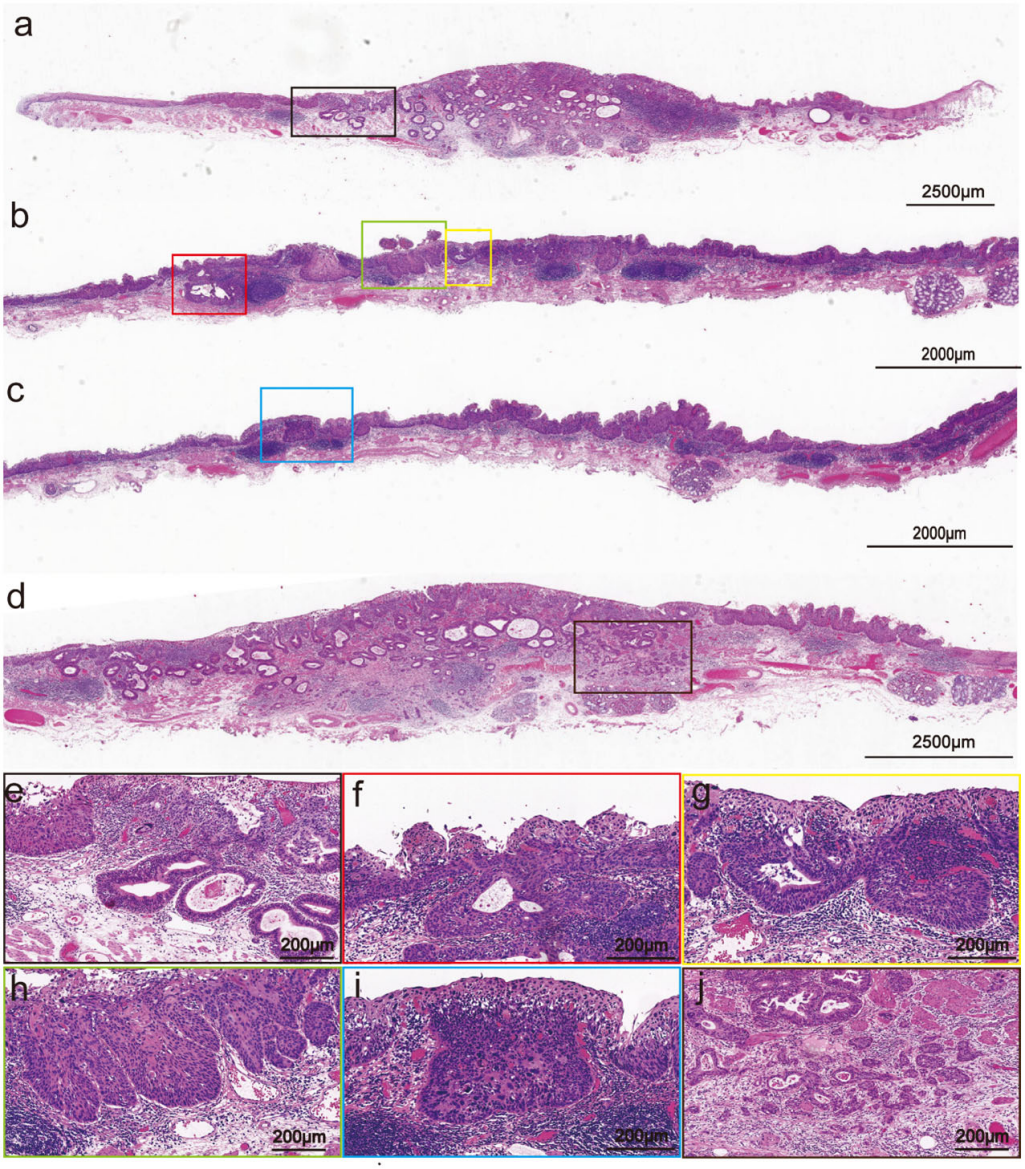
Pathohistological images of the hematoxylin and eosin-stained specimens of the resected specimen. **(a)** Variably sized tubular structures diffusely dispersed in the mucosal and submucosal layers (The scale bar represents 2500 μm). **(b, c)** The center of the tumor is poorly differentiated BSCC (Scale bars represents 2000 μm). **(d)** The he INFc tumor infiltration pattern (The scale bar represents 2500 μm). **(e)** Close-up view of the black frame in **(a)**: The tubular structure has bilayer epithelial cells (The scale bar represents 200 μm). **(f, g)** Close-up view of the red and the yellow frames in **(b)**: The tubular structure transitional with the surface squamous cell carcinoma (Scale bars represent 200 μm). **(h, i)** Close-up view of the green and the blue frames in **(b, c)**: The tumor nests arranged in a fence-like pattern and showed the BSCC differentiation (Scale bars represent 200 μm). **(j)** Close-up view of the brown frames in [**(d)**, the scale bar represents 200 μm].

A rough-surfaced type 0-IIa lesion in the center with a size of 2.7 cm×2.5 cm and unstained with Lugol’s iodine. Microscopically, the tumor was a poorly differentiated ESCC with a glandular structure (**Figure 2a**). Meanwhile, the margins of the tumor were squamous epithelial high-grade intraepithelial neoplasia (M1) and intramural invasive squamous cell carcinoma (M2). Moreover, the glandular duct openings are visible on the surface. The structural atypia of the lesion was significant. The cancer cells formed solid nests and lobules structures and round to ovoid hyperchromatic nuclei. Regional tumor nests arranged in a fence-like pattern (corresponding to the light-brown papillary-structure area under endoscopy, **Supplementary Figures 1a–c**), which showed the basaloid squamous cell carcinoma (BSCC) differentiation (**Figures 2h, i**). The percentage of basaloid components in the entire lesion was about 30%. The central of the tumor was esophageal basaloid squamous cell carcinoma (EBSCC) with tubular structures (**Figures 2b, c**), which are very distinct (consistent with the fine network pattern under endoscopy, **Supplementary Figures 1d–f**) and exhibited a bilayer-tubular differentiation. In addition, the tubular structures transitional with the surface BSCC (**Figures 2f, g**). The cell atypia of the bilayer-tubular structures was significant, and the cytoplasm of the inner cells on the lumen was rich and stained intensely red (**Figure 2e**). Moreover, the lesion showed INFc tumor infiltration pattern (infiltrating growth and an indistinct border with the surrounding tissue, **Figures 2d, j**), and flaky diffusely infiltrated into the deep layer of submucosa (T1b-SM2, depth of tumor invasion into the submucosa was 1500 µm from the muscularis mucosa).

**Figure 3:**
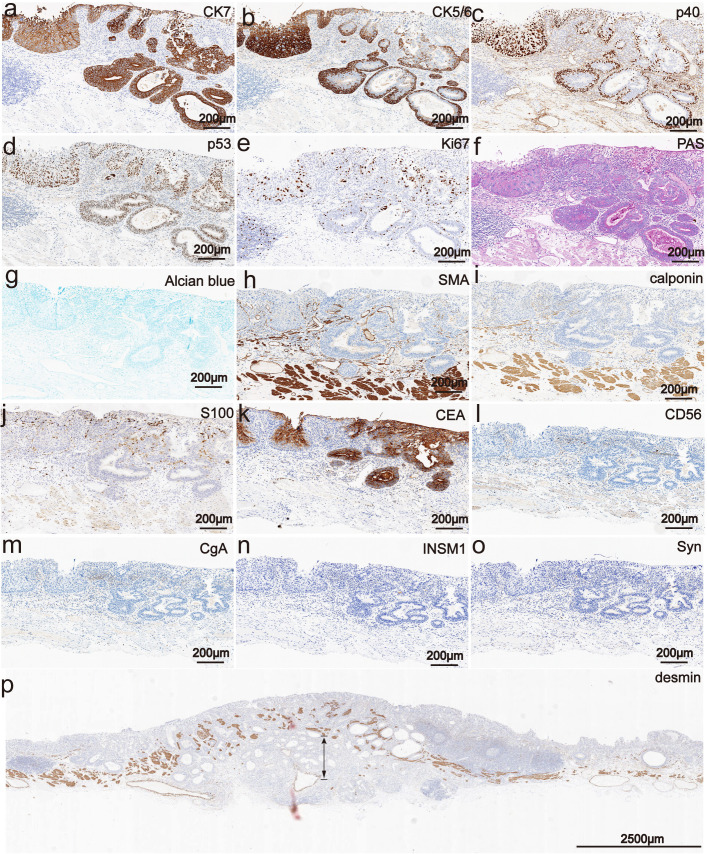
Phenotypic marker expression by immunohistochemistry staining. **(a)** The double-layer epithelium cells of the tubular structures were expression for CK7 (The scale bar represents 200 μm). **(b)** The outer cells of the tubular structures were expressed CK5/6 (The scale bar represents 200 μm). **(c)** The outer cells of the tubular structures were expressed p40 (The scale bar represents 200 μm). **(d)** p53 mutants were all positive expression in the double-layer epithelium cells and exhibited diffuse strong positive expression in the nucleus (The scale bar represents 200 μm). **(e)** The Ki-67 positive index was approximately 30% (The scale bar represents 200 μm). **(f, g)** PAS was positively expressed in the ductal differentiation components but negative expression in the basaloid cell carcinomas components, meanwhile the AB showed weak positive expression in the ductal differentiation components but negative expression in the basaloid cell carcinomas components (The scale bars represent 200 μm). **(h-j)** The SMA, calponin and S100 were all negative expression (Scale bars represent 200 μm). **(k)** The CEA were all strongly positive expression in the double-layer epithelium cells (Scale bars represent 200 μm). **(i-o)** The INSM1, CD56, CgA and Syn were negative expression in the lesion. (Scale bars represent 200 μm). **(p)** Immunohistochemically, mucosal muscle staining of desmin showed that the tubular structures diffusely infiltrated into the deep layer of submucosa (The scale bar represents 200 μm).

**Figure 4:**
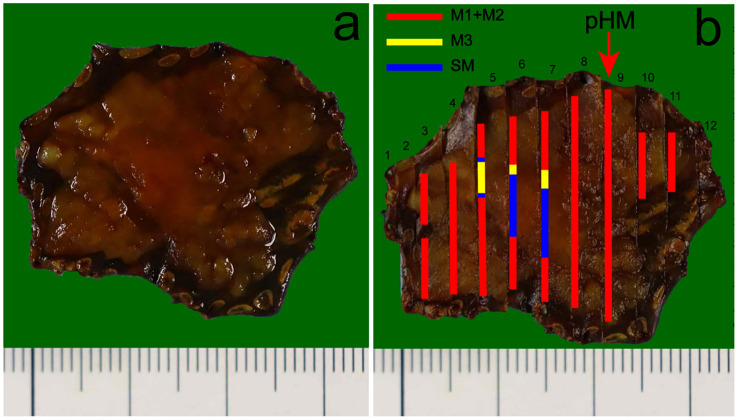
Pathological recovery diagrams. **(a)** Pathological recovery diagrams without the mapping alongside. **(b)** Early EBSCC with ductal differentiation, pT1b-SM2, Ly0, V0, (pathology horizontal margin) pHM0, (pathology vertical margin) pVM0. The cancerous lesion was 27 mm×25 mm. The red arrow indicates the location of the lesion closest to the horizontal margin of the specimen was negative. In addition, the pathology vertical margin (pVM) was also negative. The red, yellow and blue markings represent the tumor infiltrates into epithelium and lamina propria mucosae layers (M1 and M2), muscularis mucosae layer (M3), submucosa layer (SM), respectively.

Immunohistochemical results showed that the bilayer-tubular structures exhibited a bidirectional differentiation towards the outer basal epithelium and the inner columnar epithelium. The double-layer epithelium cells of the tumorous tubules expressed CK7, and the outer cells expressed p40, CK5/6 (**Figures 3a–c**). However, the SMA, calponin and S100 were all negative expression (**Figures 3h–j**). Moreover, p53 mutants (with diffuse strong positive expression in the nucleus) were found in both the outer basal epithelium and the inner columnar epithelium cells (**Figure 3d**), while negative-expression in the non-tumorous glands (**Supplementary Figure 2**). The Ki-67 positive index was approximately 30% (**Figure 3e**). The special staining of Alcian blue (AB) and periodic acid Schiff (PAS) stains are used to detect acidic mucus and neutral mucus in the same tissue. Acidic mucus is colored blue by AB, while neutral mucus is colored red by PAS (8). The results showed that the PAS was positively expressed in the ductal differentiation components but negative expression in the basaloid cell carcinomas components, meanwhile the AB showed weak positive expression in the ductal differentiation components but negative expression in the basaloid cell carcinomas components, which indicate the glandular lumina contain mucus (**Figures 3f, g**). CEA were all strongly positive expression in the double-layer epithelium cells, which further exhibited the glandular differentiation (**Figure 3k**). Moreover, the immunohistochemical staining of INSM1, CD56, CgA and Syn were negative expression in the lesion. The results suggest that there is no neuroendocrine differentiation in this tumor (**Figures 3i–o**). Mucosal muscle staining of desmin showed that the tubular structures diffusely infiltrated into the deep layer of submucosa (**Figure 3p**). Therefore, the final pathological diagnosis of the lesion was a type 0-IIa, 2.7×2.5 cm, early EBSCC with ductal differentiation, pT1b-SM2, Ly0, V0, pHM0 and pVM0 (**Figure 4**). Then the patient received a conventional radiation therapy, and at the time of manuscript submission, the patient was followed for 20 months to date without disease progression.

## Discussion

Here, we report a unique case of early esophageal squamous cell carcinoma (ESCC) with ductal differentiation. The tumor center was poorly differentiated esophageal basaloid squamous cell carcinoma (EBSCC) with a tubular structure, which showed a pattern of invasive growth and regional infiltrated into the deep layer of the submucosa. Histologically, the tubular components had a bilayer epithelial structure, exhibiting bidirectional differentiation towards the outer basal epithelium and the inner columnar epithelium.

The clinical manifestations of esophageal carcinoma (EC) with ductal differentiation are not significantly different from ordinary EC, but it has a highly malignant biological behavior. Related reports indicate that the overlying squamous cell epithelium of early EC with ductal differentiation may not exhibit significant atypia. Even in the advanced-stage, when patients experienced esophageal stenosis and progressive dysphagia, endoscopic may still only present as rough, erosive or superficial ulcer appearance. However, the postoperative pathology confirmed that the tumor has infiltrated deep into the esophagus. In the present case, the appearance of uplift and swelling caused by submucosal lesions indicated that the lesion may infiltrate into the submucosal layer. In addition, the presence of the fine network patterns suggests that this lesion may have a special glandular tubular-like components different from the ordinary ESCC. Histologically, the oral and anal sides of the lesions presented a papillary structure, consistent with the distinctive papillary structure observed under the narrow band imaging magnifying endoscopy (NBI-ME), which is often seen in gastric papillary carcinoma but rarely in ESCC. At the same time, the lesion showed tubular structures transitional with the surface squamous epithelial carcinoma from the periphery to the center, which may be the reason of the surrounding lesion exhibited an intraepithelial papillary capillary loop (IPCL) of type B1 under endoscopy, while the mixed presentation of fine network pattern and type B1 in the center. Consistent with the endoscopic results, the pathological diagnosis was early EBSCC with ductal differentiation, and the tumor invaded the muco-muscular and regionally invaded the submucosa (T1b-SM2, 1500 µm). This lesion exceeded the relative indication of endoscopic submucosal dissection (ESD). Considering the patient’s age, and high risk of trauma surgery, the patient had not chosen additional surgery, but underwent radiation therapy.

The normal esophageal ducts are located between the squamous epithelium and muscularis propria, and have a double-layer epithelial structure (9–12). The morphologies of this case are similar to the normal esophageal ducts, which displayed the outer basal epithelium cells of differentiated ducts expressed for p40, CK5/6, as well as the double-layer epithelium cells expressed CK7. However, the SMA, calponin and S100 were all negative expression. The expression of p53 protein and its variant form have important clinical significance in the diagnosis and monitoring of EC. In normal esophageal squamous epithelium, the expression of p53 protein is usually the wild-type form, showing varying levels of nuclear positivity. The expression of p53 mutant protein is very common in EC and is characterized by strong and consistent nuclear positivity, which is an important diagnostic marker of ESCC (13). Moreover, p53 mutants (with diffuse strong positive expression in the nucleus) were found in both the outer basal epithelium and the inner columnar epithelium cells.

The origin of EC with ductal differentiation is still controversial. It is unclear whether EC with ductal differentiation is an independent histological type or a special morphological type of adenocarcinoma and adeno-squamous carcinoma. The 5th edition of the World Health Organization (WHO) Classification of Digestive System Tumors classifies EC with ductal differentiation as adeno-squamous carcinoma, and suggesting that it may originate from the submucosal gland of the esophagus (5). The intrinsic glandular duct of the esophagus is composed of multiple layers of short columnar cells that open onto the surface of the esophageal epithelial layer. As a separate classification of malignant epithelial tumor of the esophagus, esophageal adenosquamous carcinoma is a tumor composed of malignant squamous and glandular epithelial components, which is different from pure ESCC.

Tamura et al. (6) reported a case of EC exclusively composed of adenocarcinoma components resembling esophageal gland ducts, and at the top of the lesion, connections were observed between the infiltrating adenocarcinoma components and the surface covered epithelium. The morphological and immunohistochemical examinations suggested that the adenocarcinoma components may arose from the esophageal surface epithelium and differentiated into an esophageal gland duct. Endoh et al. (7) also reported a case of esophageal adenocarcinoma (EAC) differentiated towards esophageal gland duct, as the covered squamous epithelial cells did not exhibit significant atypia. In the salivary glands, it is believed that undifferentiated stem cells can differentiate into squamous cells, ductal cells, myoepithelial cells or acinar cells (13). Studies have shown that uncommitted stem cells are located in the papillae of the basal layer of the esophageal epithelium (14). Therefore, some researchers speculate that the differentiated catheter may originate from esophageal undifferentiated stem cells. Moreover, the outer cells of the tumorous tubules expressed p40, CK5/6, which indicate the lesion accompanied with ESCC components. Furthermore, CK7 and CEA were all strong positive expression in the double-layer epithelium cells. which exhibited a glandular differentiation. In addition, the special staining of the PAS was positively expressed in the ductal differentiation components but negative expression in the basaloid cell carcinomas components, meanwhile the AB showed weak positive expression in the ductal differentiation components but negative expression in the basaloid cell carcinomas components, which indicate the glandular lumina contain mucus. It was further suggested that the double-layer epithelial structure was originate from the glandular ducts of the esophagus. All above results suggested that this case accompted with ductal differentiation.

It is known that basaloid squamous cell carcinoma (BSCC) has the capacity to differentiate in different ways (15). Besides, studies have confirmed that from the genetic level, the pathogenesis of EC with ductal differentiation is more similar to that of ESCC. The present case is an early poorly-differentiated EBSCC with ductal structure, manifested as a double-layer epithelial structure. There were duct opening on the surface, and the regional tumor nests are arranged in a fence-like pattern, showing an EBSCC differentiation. Moreover, the tubular structure transitional with the surface ESCC. In summary, we speculate this case may originate from the intrinsic glandular duct of the esophagus. More cases need to be accumulated and further molecular detection techniques, including next-generation sequencing (NGS) needed to further clarify the origin of such cases.

EBSCC is a rare and poorly differentiated variant of ESCC. EBSCC is derived from esophageal epithelial basal cells or undifferentiated cells with similar multipotential features (16). The cytological and histological characteristics of EBSCC are similar to normal squamous epithelial basal cells, but with a lower differential degree and worse prognosis. Histologically, EBSCC is characterized by a submucosal tumor-like growth with tumor nests invading the submucosal layer and deeper structures, and the formation of an elevated lesion (17). These lesions often covered with a non-neoplastic surface, and are difficult to diagnose accurately by endoscopy examination and biopsy. Compared to conventional ESCC, EBSCC is more frequently associated with diffuse or multifocal squamous dysplasia. Pathologically, EBSCC is considered as a highly aggressive tumors due to their propensity for early lymph node involvement, vascular invasion and recurrence (18). The prognosis of EBSCC is poorer than that of typical ESCC in advanced stages. However, in early stages, the prognosis of EBSCC does not differ significantly from that of typical ESCC (19). Therefore, early detection and diagnosis are important for improving the prognosis of EBSCC.

In conclusion, although this case is an early EC, it is accompanied by components of poorly differentiated BSCC and infiltrated into the deep layer of submucosa, which might indicate a high degree of malignancy. Further learning of the present case can help endoscopists and pathologists to improve the diagnosis and treatment level. Meanwhile, it is necessary to accumulate more case data from multiple centers to gradually establish a standardized diagnosis and treatment process for EBSCC with ductal differentiation. In addition, the specific regulatory mechanisms need to be further elucidated through more cases studies. Apart from, we recommend close follow-up of patients with these characteristics.

## Conclusion

Early esophageal basaloid squamous cell carcinoma (EBSCC) with ductal differentiation is a rare type of esophageal carcinoma (EC). It has characteristic endoscopic, morphological and immunophenotypic features, which are significantly different from conventional esophageal squamous cell carcinoma (ESCC). Furthermore, the origin of these EC requires further research.

## Data Availability

The original contributions presented in the study are included in the article/**Supplementary Material**. Further inquiries can be directed to the corresponding authors.
