# Supplementary material for: Case Report: A rare case of early esophageal basaloid squamous cell carcinoma with ductal differentiation
Source: Front Oncol. 2025 Feb 28;15:1508285. doi: 10.3389/fonc.2025.1508285 (PMC11906321; doi:10.3389/fonc.2025.1508285)
Supplement: Supplementary file 1 [file DataSheet1.docx]

# Supplementary Figures


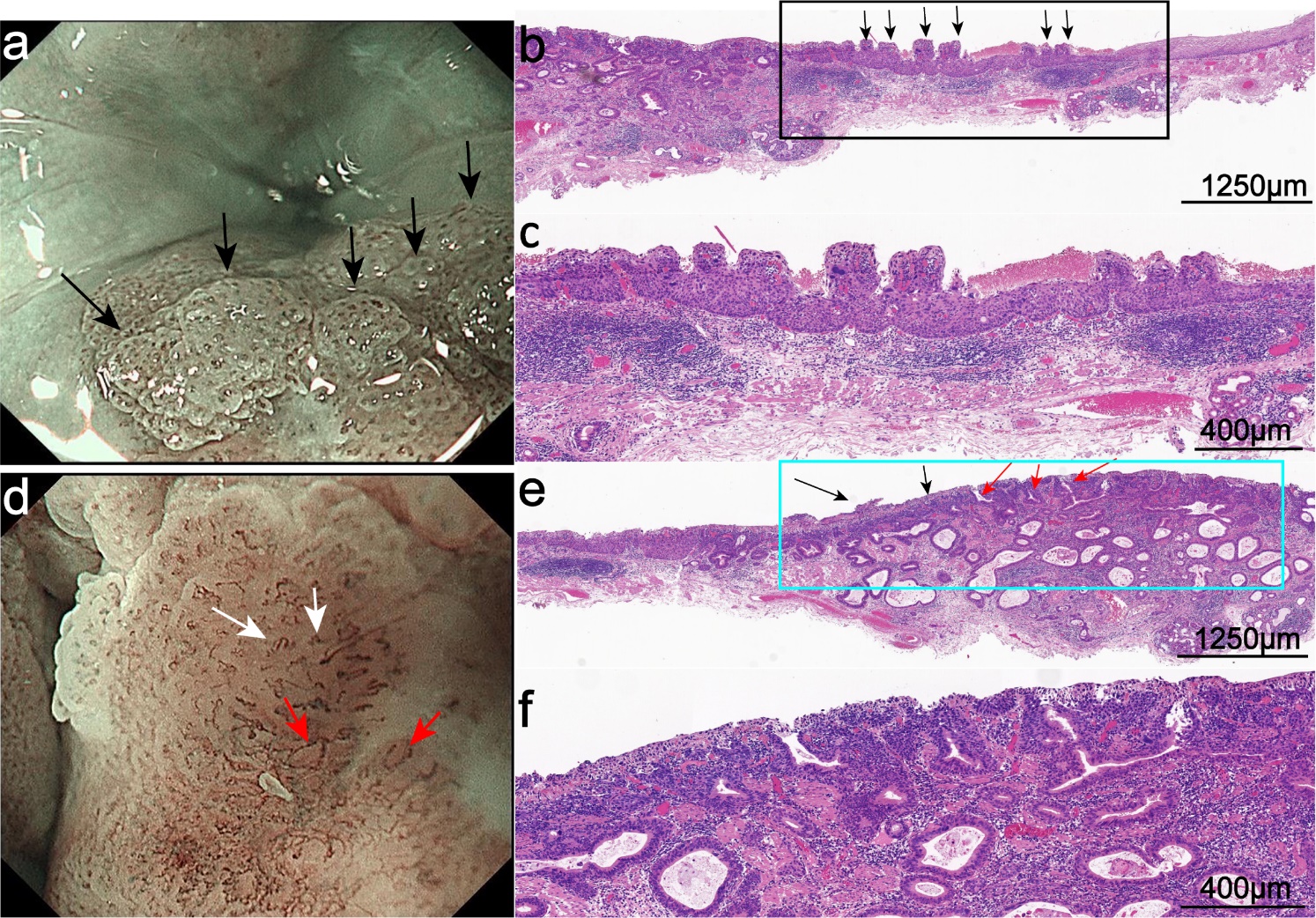


**Supplementary Figure 1.** Contrastive analysis for the endoscopic images and pathohistological images of the resected specimen. (**a**) The NBI-ME showed that the papillary-like structures of the lesion (Black rows). (**b**) Papillary-like structures (Black rows) consistent with the structure observed in NBI-ME in (a) (The scale bar represents 1250μm). (**c**) Close-up view of the black frame in (b) (The scale bar represents 400μm). (**d**) The fine network pattern of microvascular (Red arrows) and the IPCL of type B1(White arrows). (**e**) Tubular structures (Red rows) transitional with the surface squamous epithelial carcinoma (Black rows) from the periphery to the center (The scale bar represents 1250μm). (**f)** Close-up view of the blue frame in (e) (The scale bar represents 400μm).


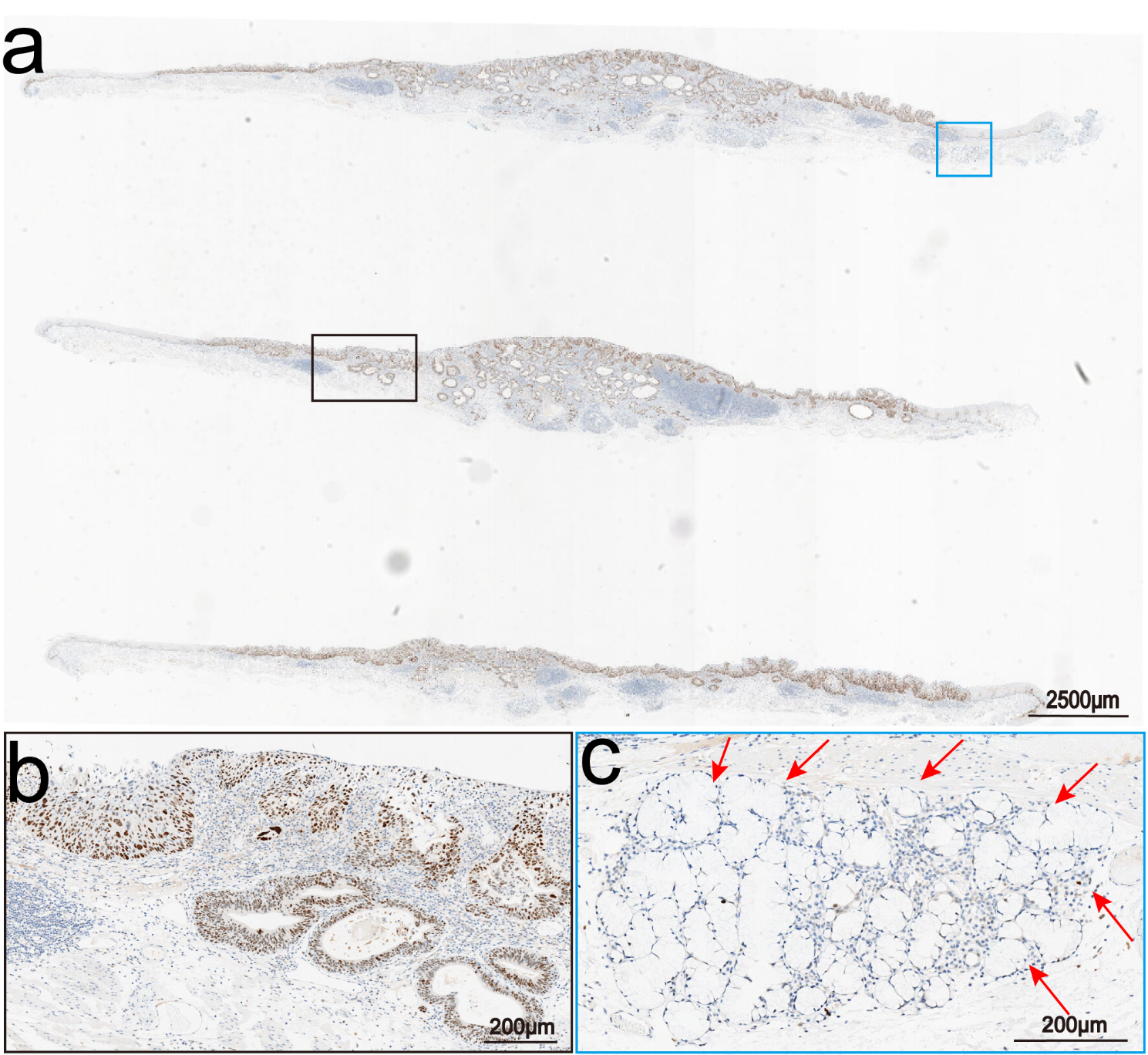


**Supplementary Figure 2.** Phenotypic marker expression by immunohistochemistry staining of P53. (**a**) Variably sized tumorous glands and non-tumorous glands diffusely dispersed in the mucosal and submucosal layers (The scale bar represents 2500 μm). (**b**) Close-up view of the black frames in (**a**), P53 was positive-expression in the tumorous glands (The scale bar represents 200 μm). (**c**) Close-up view of the blue frames in (**a**), P53 was negative-expression in the non-tumorous esophageal glands (Red arrows, the scale bar represents 200 μm).
